# Dislocation of the Knee Joint, with Fracture of the Patella

**Published:** 1855-09

**Authors:** B. O. Jones

**Affiliations:** Atlanta, Georgia


ARTICLE IV.—*Dislocation of the Knee Joint with Fracture of the Patella.* By B. O. JONES, M. D., Atlanta, Georgia.

W. E. P., aged 35 to 40 years, of rather stout constitution, subject to an occasional spree, was thrown from a horse, in full speed, during intoxication, and falling on a large rock, sustained considerable damage, among others a compound dislocation of the knee joint with fracture of the patella.

When seen by me, immediately after the accident, he presented the following symptoms: Large lacerated wound of the common integuments on the internal portion of the knee; fracture of the patella; dislocation of the joint laterally and inclining backwards, rupturing the ligaments, and producing dreadful deformity; the leg nearly at a right angle with the thigh, discharging blood, and as I then thought, synovial fluid, freely presenting on a thorough examination altogether a wound of such magnitude that I was greatly at a loss what course to pursue for the best. Being at some distance from any consulting physician of much eminence, I concluded to reduce the dislocation, dress the parts, remove the patient home, which was but a short distance, and wait, as it was late at night, until the morning, before determining what further to do. When morning came I found my patient quite calm and composed, free from intoxication and perfectly submissive, except as to my sending for consulting physicians, saying that he would not by any means have his leg amputated, if it was thought best even by a host of physicians, preferring rather that if die he must, for all to go together; that he greatly preferred that I should do the best I could in the premises, and trust to a "Higher Power" for the perfection of the cure, (or words to that effect,) which induced me to proceed, unassisted, in the case, although I must admit I had but little hope of success.

Treatment was quite simple, I must confess. Under an impression that the case must prove fatal, and that, too, in a few days, I concluded not to annoy the patient with much restraint, preferring, as there was little prospect of recovery, to *smooth* the road to death. I put him under the full influence of anodynes, and after adjusting carefully the dislocation, and approximating the fractured extremities of the patella, replacing the integuments, &c., I applied the common starch bandage, with pasteboard splints, making an opening a day or so thereafter in the bandage, over the wound, in the soft parts, which bandage and dressing remained for some considerable time longer, *greatly longer*, than bandages of that sort are usually permitted to remain, for the simple reason that my patient improved so fast—"getting well," as he would say, so astonishingly fast, that I concluded to let everything stand as it was, unless some change for the worse, then to act as occasion should require or symptoms demand.

These, with antiphlogistics, such as saline cathartics, &c., constituted the principal treatment in the case, and in the course of some five or six weeks I had the satisfaction to see my patient greatly improved—able to be out of bed, and to my astonishment, in removing the dressing, found that the joint was not immovable, but apparently much stiffened and greatly enlarged, presenting more the appearance, than otherwise, that condition which follows inflammation of the knee joint, wherein suppuration was prevented by the use of local applications, such as blisters, &c.

To conclude this hasty sketch, I have to state, although I know it will appear unreasonable, that the patient continued gradually to improve until now, several years since the accident, he suffers no apparent inconvenience whatever from the injury; has the entire use of the joint, free from any seeming deformity or lameness; and what is most remarkable of all, especially in a "temperance" point of view, it utterly failed to make a sober man of him, leaving him still the devotee of the fiery god, yet with a heart as generous, and a soul as broad, as the universe.

Thus will be seen a case which would most clearly to my mind indicate the use of the knife, in which nature, assisted by art, performed the cure, and that, too, in a manner so beautiful, prompt, and efficiently, that I, with many others acquainted with the history of the case, was constrained to express my gratitude to Providence for the result.

I would have preferred more time to get up this case for publication; being, as you know, unaccustomed to write for publication, I fear there will be many inaccuracies; besides, having to write from memory, I cannot state the facts as clearly as I should have desired. Yet I hope the case may answer some useful purpose—if so, I shall have accomplished one great object of my heart's desire—the advancement of the healing art. Should I fail in this, it will at least manifest in an humble way my readiness to promote the prosperity of the enterprise which has for its objects, as all will admit, the advancement of science and the amelioration of suffering humanity.

